# The mood disorder spectrum vs. schizophrenia decision tree: EDIPHAS research into the childhood and adolescence of 205 patients

**DOI:** 10.1186/s12888-022-03835-0

**Published:** 2022-03-18

**Authors:** Mathilde Léger, Vanessa Wolff, Bernard Kabuth, Eliane Albuisson, Fabienne Ligier

**Affiliations:** 1 Pôle Universitaire de Psychiatrie de l’Enfant et de l’Adolescent [University Department of Childhood and Adolescent Psychiatry], Centre Psychothérapique de Nancy [Nancy Psychotherapy Centre], F-54520 Laxou, France; 2 EA 4432, PRISME, Université de Lorraine [Lorraine University], Laxou, France; 3 DRCI UMDS, Centre Hospitalier Universitaire de Nancy, Nancy University Hospital, Laxou, France; 4 EA 4360 APEMAC, Université de Lorraine, Laxou, France

**Keywords:** Adolescence, Mood spectrum disorder, Decision tree, Schizophrenia, Trajectory

## Abstract

**Background:**

The early detection of patients at risk of developing schizophrenia and bipolar disorder, and more broadly mood spectrum disorder, is a public health concern. The phenotypical overlap between the prodromes in these disorders calls for a simultaneous investigation into both illness trajectories.

**Method:**

This is an epidemiological, retrospective, multicentre, descriptive study conducted in the Grand-Est region of France in order to describe and compare early symptoms in 205 patients: 123 of which were diagnosed with schizophrenia and 82 with bipolar disorder or mood spectrum disorder. Data corresponding to the pre-morbid and prodromal phases, including a timeline of their onset, were studied in child and adolescent psychiatric records via a data grid based on the literature review conducted from birth to 17 years of age.

**Results:**

Two distinct trajectories were highlighted. Patients with schizophrenia tended to present more difficulties at each developmental stage, with the emergence of negative and positive behavioural symptoms during adolescence. Patients with mood spectrum disorder, however, were more likely to exhibit anxiety and then mood-related symptoms. Overall, our results corroborate current literature findings and are consistent with the neurodevelopmental process. We succeeded in extracting a decision tree with good predictability based on variables relating to one diagnosis: 77.6% of patients received a well-indexed diagnosis. An atypical profile was observed in future mood spectrum disorder patients as some exhibited numerous positive symptoms alongside more conventional mood-related symptoms.

**Conclusion:**

The combination of all these data could help promote the early identification of high-risk patients thereby facilitating early prevention and appropriate intervention in order to improve outcomes.

## Background

The division of psychiatric disorders into “dementia praecox”, also known as schizophrenia, and “manisch-depressives Irresein” in German, now conceptualised as bipolar disorder, was first proposed by Emil Kraepelin in 1899. Both are severe, chronic, psychiatric disorders that remain significant public health concerns to this day. Taking the full spectrum of mood disorders into consideration, they affect up to 4.4% of the population and rank 4^th^ in terms of global causes of morbidity and mortality among people under 25 years of age [1]. Schizophrenia affects between 0.3 and 0.7% of the global population [2]. Recovery is mostly partial but may become debilitating [3].

There remains a significant delay of approximately 10 years in the diagnosis of bipolar disorder [4]. Yet, we know that this condition can have devastating consequences if left untreated [5–7]. Schizophrenia is also a diagnosed late [8] and is linked to greater severity, poorer remission and a greater risk of relapse [9]. The efficacy of early intervention has been established with reduced transition towards psychosis [10, 11]. Consequently, the early detection of these conditions is a key objective.

In child and adolescent psychiatry, determining what the future holds for young patients presenting psychotic or mood-related symptoms can be a sensitive issue. A comprehensive understanding of the early signs and illness trajectories of schizophrenia and mood spectrum disorder is vital for personalised prevention and treatment strategies. Recent studies promote a neurodevelopmental model with a genetic overlap between both disorders [12], common risk factors [13] and shared cognitive impairment [14]. Nevertheless, additional factors adversely affecting neurodevelopment may impact patients with schizophrenia compared to those who go on to develop bipolar disorder [15, 16].

A prodromal phase has been identified in both pathologies, preceding the onset of the disorder *per se* by several months. An initial pre-morbid phase has also been described [1, 17]. These phases are now clearly defined in schizophrenia [18] using scales and questionnaires to identify individuals at high risk of psychosis [19]. Mood-related symptoms are also known to affect the development of bipolar disorders [20, 21]. However, these symptoms are not pathognomonic and the phenotypical overlap between the prodromes of schizophrenia and mood spectrum disorders requires simultaneous investigation of both illness trajectories.

It is interesting to compare these symptoms more precisely, focusing on connections and timing in relation to the onset of both disorders.

## Methods

### Objective

An objective study involves the construction of a decision tree to provide a descriptive, comparative analysis of the symptoms and a timeline of their onset during the childhood and adolescence of patients with schizophrenia or mood spectrum disorder.

### Study design

This is an epidemiological, retrospective, multicentre, descriptive study conducted within the EDIPHAS (Dimensional Study of The Early Phases of Affective Disorders and Schizophrenia) research project framework [22]. Public psychiatric hospitals located in the Grand Est region of France served as recruitment centres for this study.

The eligibility criteria were as follows:

- Adult psychiatric follow-up received between 2006 and 2017
- Diagnosis of schizophrenia (F20) or mood spectrum disorder: F30 (manic episode), F31 (bipolar disorder), F33 (recurrent major depressive disorder), F34 (persistent mood disorder) according to ICD10
- Between 18 and 30 years of age at the time of adult psychiatric follow-up

The exclusion criteria were as follows:

- No previous childhood or adolescent psychiatric follow-up before the age of 18 and prior to diagnosis
- Refusal to participate in the study.

### Material

Adult psychiatric records were accessible only via computer software and were studied in order to establish the age at diagnosis. Data concerning childhood and adolescent symptoms were collected from the patients’ child and adolescent psychiatric medical records. They were stored in a data grid compiled following a literature review. This tool demonstrated satisfactory inter-rater reliability in previous EDIPHAS studies [22]. The patient’s age at time of onset was documented for each item. Symptoms were grouped according to clinical dimensions: cognitive/developmental, functional, anxiety-related, behavioural/impulse control, mood-related, negative and positive/discordant symptoms.

### Population

Eligible study subjects were contacted by a letter sent either to their place of residence or to the then referring psychiatrist. This letter contained clearly set out the study objectives. It also stated that patients were entitled to refuse any requests to access or review their earlier medical records without their negative decision adversely impacting their treatment.

### Analysis

Qualitative variables were represented in terms of both absolute number and population percentage. Quantitative variables were represented by average and standard deviation. The Mann-Whitney U-test, Chi2 or Fisher’s exact test were also applied depending on the nature and distribution of the variables. CHAID (Chi-squared Automatic Interaction Detection), which is a multivariate approach, was used to perform classification and segmentation analysis. The decision tree was constructed by repeatedly splitting a node (group) into two or more nodes, beginning with the entire data set. At each step, the predictor (covariable) that gives the best prediction is selected by the model and the node is split into two or more nodes based on the predictor values. SPSS has extended the algorithms to handle nominal, categorical, ordinal and continuous target variables. Chi square statistics were used to identify optimal splits. CHAID with crossed validation was used to build a decision tree with adult schizophrenia and mood spectrum disorder diagnoses as the outcome. We selected the covariables for the multivariable analysis (CHAID) based on significant results in univariate analysis, clinical reasoning and previous studies.

In the analysis, the level of significance was α=0.05.

IBM SPSS Statistics v22 software was used for the statistical analysis.

## Results

### General and clinical description of the population

Four hundred and fifty-three (453) adult patients were identified as eligible: 324 had been diagnosed with mood spectrum disorder (MSD) and 129 with schizophrenia (SZ). Among these patients, 242 did not have paediatric psychiatric records. Two sets of records did not contain information prior to diagnosis, 2 did not contain enough information for assessment and 2 patients declined to participate. Finally, 205 patients were enrolled in the study - 82 with MSD and 123 with SZ.

The SZ population mostly comprised males (67.5%) born during the winter (33.3%), while the MSD population consisted of mainly females (71.6%) born during the spring (40.2%). Only a few of the records contained detailed obstetric information. Foetal injury or neonatal complication was documented for 9.8% of SZ patients and 12.2% of MSD patients. In terms of family history, there was a high incidence of psychiatric illness amongst first degree relatives: 55.3% for SZ patients and 57.3% for MSD patients. In the majority of cases, the family history revealed mood disorders (39% in MSD and 25.2% in SZ) followed by psychosis (13.8% in SZ and 6.1% in MSD). A family history of suicide attempts was documented in 6.5% of the SZ population versus 0% of the MSD population. The average age of diagnosis was 20.6 years (Standard Deviation SD=3.9) in the SZ population and 20.9 years (SD=4.1) in the MSD population.

**Fig. 1:**
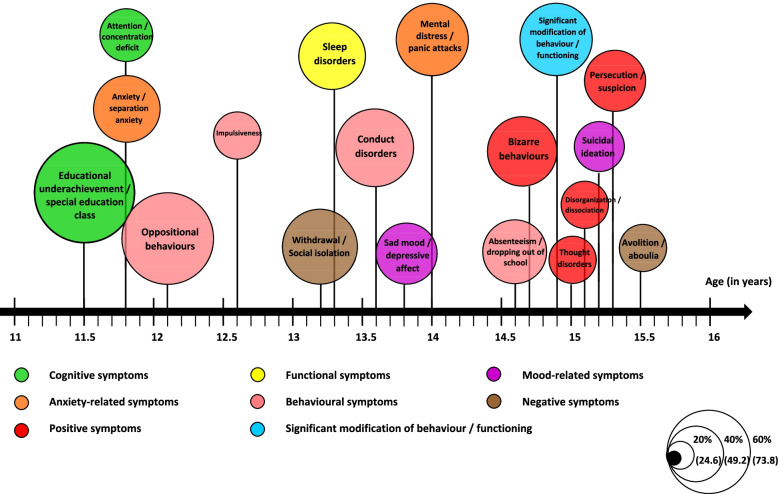
Clinical trajectory during childhood and adolescence for a population of 123 adults presenting schizophrenia; selection of the most typical symptoms >/= 41 patients (33%); the size of the circles is proportional to the number of patients with the corresponding symptoms

**Fig. 2:**
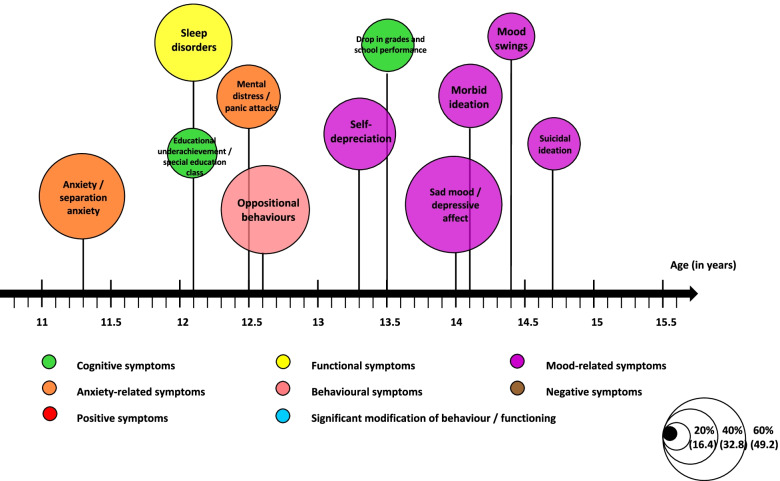
Clinical trajectory during childhood and adolescence for a population of 123 adults suffering from mood spectrum disorders; selection of the most representative symptoms >/= 27 patients (33%); the size of the circles is proportional to the number of patients with the corresponding symptom

Some symptoms were presented by more than one-third of patients and tended to emerge in a particular sequence. Trajectory modelling appears to be an interesting approach for both populations in order to summarise the natural development of symptoms and the average age of onset. This information is presented in Figures 1 and 2.

### Comparison of populations

Only one variable, namely the age of onset of “relational disturbances involving inhibition”, was not evenly distributed in both diagnostic categories (Mann-Whitney U test = 0.028).

Overall, 41 variables were significantly linked to diagnosis during independence tests (p<0.05). More symptoms were associated with the diagnosis of SZ than MSD. Detailed results according to each dimension are shown in Tables 1, 2, 3 and 4.

**Table 1:** Cognitive and developmental symptoms in the childhood psychiatric history of adults presenting schizophrenia and mood spectrum disorder (*n*=205)

	Schizophrenia *n*=123		Mood spectrum disorder *n*=82		Chi2/Fisher
Symptoms	Number of Patients (% of the population)	Age at Onset (in years) Mean (SD)	Number of Patients (% of the population)	Age at Onset (in years) Mean (SD)°	*p* value
**Cognitive and developmental symptoms**					
Low IQ (<69)	24 (19.5%)	-	0 (0%)	-	<0.0001*
High IQ (>120)	1 (0.8%)	-	2 (2.4%)	-	0.565
Heterogeneous or discordant IQ	12 (9.8%)	-	1 (1.2%)	-	0.014*
Delayed psychomotor development	17(13.8%)	-	7 (8.5%)	-	0.249
Lateralisation disturbances	2 (1.6%)	-	1 (1.2%)	-	1.000
Time and space orientation disturbances	14 (11.4%)	-	5 (6.1%)	-	0.071
Other psychomotor disturbances	12 (9.8%)	-	9 (11%)	-	0.778
Speech/language disorders and delay	21 (17.1%)	-	7 (8.5%)	-	0.081
Articulation disorders	5 (4.1%)	-	4 (4.9%)	-	1.000
Written language disorders	13 (10.6%)	-	9 (11%)	-	0.927
Mathematics disorders	9 (7.3%)	-	4 (4.9%)	-	0.483
Learning disorders not otherwise specified	11 (8.9%)	-	11 (13.4%)	-	0.311
Drop in grades and school performance	63 (51.2%)	14.3 (2.6)	32 (39%)	13.5 (3)	0.086
Educational underachievement or special educational needs	89 (72.4%)	11.5 (4.5)	30 (36.6%)	12.1 (4)	<0.001*
Attention and concentration deficit	46 (37.4%)	11.7 (4.6)	26 (31.7%)	13.6 (3.4)	0.403
Psycho-affective immaturity	25 (22.8%)	12.3 (3.9)	23 (28%)	12.4 (4.3)	0.391
Tics	13 (10.6%)	11.1 (3.7)	3 (3.7%)	11 (5.2)	0.071
Muteness	16 (13%)	12.9 (4.1)	4 (4.9%)	9 (3.6)	0.055
Stuttering	3 (2.4%)	11.3 (6.4)	0 (0%)	-	0.276

Legends: *SD *Standard Deviation; * significant result with *p*<0.05; the Chi2 or Fisher’s exact test was applied

**Table 2:** Functional and anxiety-related symptoms in the childhood psychiatric history of adults presenting schizophrenia and mood spectrum disorder (*n*=205)

	Schizophrenia *n*=123		Mood spectrum disorder *n*=82		Chi2/Fisher
Symptoms	Number of Patients (% of the population)	Age at Onset (in years) Mean (SD)	Number of Patients (% of the population)	Age at Onset (in years) Mean (SD)°	*p* value
**Functional symptoms**					
Anorexia	35 (28.4%)	13.1 (4.9)	23 (28%)	13 (4.9)	0.799
Binge eating disorder	7 (5.7%)	13.4 (2.3)	9 (11%)	11.8 (4.6)	0.167
Polydipsia	3 (2.4%)	16.7 (0.6)	0 (0%)	-	0.276
Bulimia	2 (1.6%)	9.5 (9.2)	10 (8.1%)	13.6 (4.3)	0.004*
Other eating disorders	10 (8.1%)	10 (6.4)	15 (18.2%)	9.3 (6.3)	0.029*
Sleep disorders	60 (48.8%)	13.3 (4.7)	46 (56.1%)	12.1 (5.1)	0.304
Parasomnias	27 (22%)	14 (3.2)	21 (25.6%)	12.9 (5)	0.545
Enuresis	16 (13%)	7.3 (3.2)	7 (8.5%)	4.7 (2.7)	0.320
Encopresis	8 (6.5%)	5.6 (2.9)	0 (0%)	-	0.023*
**Anxiety-related symptoms**					
Anxiety/separation anxiety	59 (48%)	11.7 (4.9)	52 (63.4%)	11.3 (4.9)	0.030*
Mental distress/panic attacks	64 (52%)	14 (3.1)	38 (46.3%)	12.5 (4.2)	0.425
Obsessional elements	23 (18.7%)	12.5 (4.1)	7 (8.5%)	9 (5)	0.044*
Psychosomatic symptoms	25 (20.3%)	14 (3.1)	23 (28%)	15 (2)	0.201
Phobic elements	31 (25.2%)	13.8 (4.1)	4 (4.9%)	14.7 (2.1)	<0.0001*
Anxiety-related school refusal	20 (16.3%)	14.1 (3.7)	8 (9.8%)	15.5 (1.7)	0.184

Legends: *SD *Standard Deviation; * significant result with *p*<0.05; the Chi2 or Fisher’s exact test was applied

**Table 3:** Behavioural/impulse control and mood-related symptoms in the childhood psychiatric history of adults presenting schizophrenia and mood spectrum disorder (n=205)

	Schizophrenia *n*=123		Mood spectrum disorder *n*=82		Chi2/Fisher
Symptoms	Number of Patients (% of the population)	Age at Onset (in years) Mean (SD)	Number of Patients (% of the population)	Age at Onset (in years) Mean (SD)°	*p* value
**Behavioural and impulse-control symptoms**					
Self-harm behaviours without suicidal intent	34 (27.6%)	14.2 (3.3)	26 (31.7%)	15.4 (1.5)	0.531
Self-harm behaviours with suicidal intent	28 (22.8%)	15 (3.2)	24 (29.2%)	14.7 (1.8)	0.235
Oppositional behaviours	82 (66.7%)	12.1 (4.1)	53 (64.6%)	12.6 (4.5)	0.764
Conduct disorders	70 (56.9%)	13.6 (3)	27 (32.9%)	14.1 (2.8)	0.001*
Risky behaviour	21 (17.1%)	14.4 (2.1)	20 (24.4%)	14.7 (3)	0.199
Psychomotor agitation without mood component	39 (31.7%)	12.5 (4.4)	12 (14.6%)	12.8 (4.5)	0.006*
Impulsiveness	41 (33.3%)	12.6 (4.3)	21 (25.6%)	14.7 (2.8)	0.238
Running away from home	34 (27.6%)	14.7 (2.9)	13 (15.9%)	15.2 (1.1)	0.049*
Tobacco use	28 (22.8%)	15.6 (1.5)	8 (9.8%)	15.1 (1.5)	0.016*
Substance abuse	40 (32.5%)	15.7 (1.2)	24 (29.3%)	15.2 (1.6)	0.623
Absenteeism/dropping out of school	57 (46.3%)	14.7 (3)	20 (24.4%)	15 (3)	0.01*
**Mood-related symptoms**					
Sad mood, depressive affect	54 (43.9%)	13.8 (3)	58 (70.7%)	13.8 (3.4)	<0.0001*
Psychomotor retardation	28 (22.8%)	15.5 (2)	11(13.4%)	15.2 (2)	0.095
Morbid ideation	33 (26.8%)	14.4 (2.8)	39 (47.6%)	14.1 (2.5)	0.002*
Self-depreciation	39 (31.7%)	13.5 (3.8)	43 (52.4%)	13.3 (3.3)	0.003*
Anhedonia	17 (13.8%)	15.9 (1.1)	21 (25.6%)	15.4 (1.6)	0.033*
Suicidal ideation	46 (37.4%)	15.3 (1.6)	32 (39%)	14.8 (2.3)	0.814
Mood swings	18 (14.6%)	14.3 (3.5)	29 (35.4%)	14.4 (3.2)	0.001*
Psychomotor agitation with mood component	23 (18.7%)	14 (3.9)	12 (14.6%)	13.3 (3.1)	0.049

Legends: *SD *Standard Deviation, * significant result with *p*<0.05; the Chi2 or Fisher’s exact test was applied

**Table 4:** Negative and positive symptoms in the childhood psychiatric history of adults presenting schizophrenia and mood spectrum disorder (n=205)

	Schizophrenia *n*=123		Mood spectrum disorder *n*=82		Chi2/Fisher
Symptoms	Number of Patients (% of the population)	Age at Onset (in years) Mean (SD)	Number of Patients (% of the population)	Age at Onset (in years) Mean (SD)°	*p* value
**Negative symptoms**					
Withdrawal/social isolation	66 (53.7%)	1.,2 (4.4)	26 (31.7%)	13.2 (3.9)	0.002*
Poverty of speech	42 (34.1%)	14.7 (2.5)	5 (6.1%)	15.6 (1.3)	<0.0001*
Emotional coldness	34 (27.6%)	15.4 (1.6)	12 (14.6%)	16 (1.3)	0.029*
Relational disturbances involving inhibition	39 (31.7%)	10.7 (4.9)	24 (29.3%)	7.9 (4.8)	0.711
Reluctance/refusal of contact	32 (26%)	14.6 (2.7)	13 (15.9%)	15.1 (2.6)	0.085
Avolition/aboulia	44 (35.8%)	15.5 (1.6)	6 (7.3%)	16.2 (1)	<0.0001*
Self-neglect	18 (14.6%)	14.4 (2.1)	3 (3.7%)	12.7 (4.9)	0.011*
Stereotypies	25 (20.3%)	11.5 (5.6)	0 (0%)	-	<0.0001*
**Positive and discordant symptoms**					
Bizarre behaviours	62 (50.4%)	14.7 (2.9)	10 (12.2%)	15.8 (1.2)	<0.0001*
Disorganisation, dissociation	42 (34.1%)	15.1 (2.2)	6 (7.3%)	16 (1.3)	<0.0001*
Atypical phobias	6 (4.9%)	13 (5.3)	0 (0%)	-	0.083
Fragmentation anxiety, fragmentation	14 (11.4%)	13.9 (3.7)	3 (3.7%)	14.7 (0.6)	0.049*
Thought disorders	43 (35%)	15 (2.2)	3 (3.7%)	15.7 (1.5)	<0.0001*
Confabulation/invasive imagery	16 (13%)	14.3 (3)	2 (2.4%)	15.5 (0.7)	0.009*
Delusional ideation	39 (31.7%)	15.1 (2.1)	6 (7.3%)	16 (1.1)	<0.0001*
Derealisation/depersonalisation	20 (16.3%)	14.9 (2.1)	5 (6.1%)	16 (1.4)	0.029*
Auditory hallucinations	33 (26.8%)	15.3 (1.4)	7 (8.5%)	15.3 (1.4)	0.001*
Visual hallucinations	25 (20.3%)	14.6 (2.1)	5 (6.1%)	15.4 (1.5)	0.005*
Cenesthetic hallucinations	10 (8.1%)	15.3 (2.8)	1 (1.2%)	15	0.053
Persecution, suspicion	55 (44.7%)	15.3 (1.6)	12 (14.6%)	15.3 (2.7)	<0.0001*
Other delusions	4 (3.3%)	15.5 (1.3)	2 (2.4%)	16 (1.4)	1.000
**Other symptoms**					
Significant modification of behaviour/functioning	64 (52%)	14.9 (1.8)	19 (23.1%)	14.2 (2.2)	<0.0001*
Diagnosis of attention deficit hyperactivity disorder	3 (2.4%)	11 (3.5)	2 (2.4%)	11 (5.7)	1.000

Legends: *SD *Standard Deviation, * significant result with *p*<0.05; the Chi2 or Fisher’s exact test was applied

### Decision tree

**Fig. 3:**
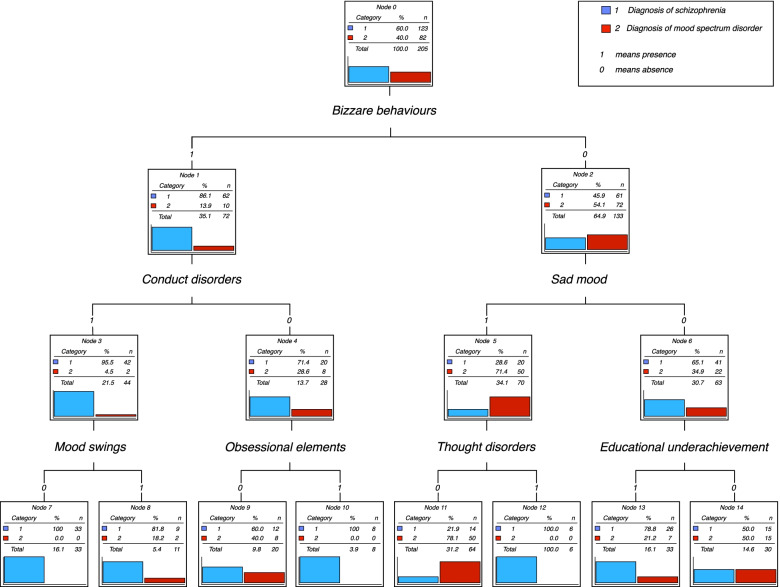
Decision tree highlighting the diagnosis of schizophrenia and a diagnosis of mood spectrum disorder during adulthood

A decision tree based on symptoms significantly associated with each diagnosis has been extracted (Figure 3). The first node represents bizarre behaviours as a result of which the tree splits into multiple edges. There are six internal nodes before the leaves. The second level includes conduct disorders and sad mood while the third is based on mood swings, obsessional elements, thought disorders and educational underachievement.

This tool offers good predictability in our population as 77.6% of the patients received the correct diagnosis. 109 of the 123 diagnosed cases of schizophrenia, (88.6%) were clearly identified. Out of the 82 diagnoses of mood spectrum disorder, 50 (61%) were clearly identified. Therefore 14 cases of schizophrenia and 32 cases of mood spectrum disorder received the wrong predictive diagnosis.

CHAID (Chi-squared Automatic Interaction Detection) is a decision tree constructed by repeatedly splitting a node (group) into two nodes, beginning with the entire data set. At each step, the predictor (covariable) that gives the best prediction is selected and the node is split into two nodes based on the predictor values (0 absence or 1 presence). The best predictor for the entire data set is ‘Bizarre behaviours’ with adjusted *p*=0.0001. The next level includes ‘Conduct disorders’ with adjusted *p*=0.004 and ‘Sad Mood’ with adjusted *p*=0.0001. This is followed by ‘Mood swing’ with adjusted *p*=0.012; ‘Obsessional elements’ with adjusted *p*=0.034; ‘Thought disorders’ with adjusted *p*=0.000 and ‘Educational underachievement’ with adjusted *p*=0.017. For example, in the absence of ‘Bizarre behaviours’, presence of ‘Sad mood’ and absence of ‘Thought disorders’, a node (group or profile) of 50 was identified out of 82 initial bipolar disorder diagnoses (61%) and 14 out of 123 initial schizophrenia diagnoses (11%).

## Discussion

### Comparison of both populations and the decision tree

We succeeded in extracting a decision tree offering good predictability in our population as 77.6% of the patients received the correct diagnosis.

All variables, apart from one, were found to be evenly distributed across both diagnostic categories. This finding may seem disappointing but it highlights the specific difficulties encountered. We harnessed symptoms characterising childhood and adolescence, and drew distinct trajectories between patients developing SZ and MSD. However, the time distribution was not specific or pathognomonic of one diagnosis. Hence the early identification and differentiation of high-risk profiles is complex.

Our study does not feature the conventionally high rate of comorbid ADHD diagnoses. Up to 58% of children with ADHD are later diagnosed with schizophrenia [23] and between 50 and 80% of those are further diagnosed with bipolar disorder [24]. Only 2.4% of the diagnoses in each group accounted for ADHD. The difference might be explained by the retrospective research involving medical records at a time when such diagnoses were not commonplace in France. Moreover, many consultations took place during adolescence, a time when undiagnosed ADHD may have been classified as behavioural disorders and educational difficulties. With reference to the MSD population in particular, it should be noted that the subjects were mostly female, and the diagnosis of ADHD is less accessible and generally delayed for women given the lack of hyperactivity.

Surprisingly, behavioural/impulse control-related symptoms (p<0.05) were linked only to the SZ diagnosis. More predictably, mood-related symptoms were associated with the MSD diagnosis. Since oppositional behaviours and mood-related symptoms are known to typically appear during adolescence in the general population [25], it is crucial to pay specific attention to their evolution and interaction with other symptoms. This approach could reveal a high-risk profile tending towards SZ or MSD. A significant correlation was noted between functional symptoms and MSD diagnosis, with the exception of encopresis, which was linked to SZ. It has also been suggested that enuresis could be indicative of predisposition towards schizophrenia [26]. Among anxiety-related symptoms, anxiety/separation anxiety was linked to MSD but phobic and obsessional elements were associated with SZ. It is important to note the impact of growing up with a mentally ill parent on the development of psychiatric disorders such as anxiety, as evidenced in several studies [27, 28]. Sixteen salient positive and negative symptoms were observed. They were linked solely to the diagnosis of SZ with significant changes in terms of behaviour or function.

These results also echo the findings of a study comparing axis I antecedents before the age of 18, namely unipolar depression, bipolar disorder and schizophrenia [29]. Compared to unipolar depression, schizophrenia was significantly associated with ADHD, a disorder connected with cognitive and behavioural symptoms in our study, and primary nocturnal enuresis. There were also significant links between schizophrenia and social phobia and the ADHD inattentive subtype compared to bipolar disorder.

The decision tree failed to give the appropriate predictive diagnosis in 46 cases out of 205. It is obvious that most of the medical records in question contained very few significant data given the succinct observations or brief follow-up. Moreover, the symptoms were frequently identified more than five years (sometimes ten) before a diagnosis was made. We can assume that they were probably associated primarily with the pre-morbid as opposed to the prodromal phase, which is recognised as being less specific. However, eight SZ cases did not meet these criteria as they exhibited symptoms relating to virtually all dimensions over time. These included mood-related symptoms, which were strongly correlated with MSD diagnosis, and a few positive symptoms. Among the SZ cases given the incorrect predictive diagnosis, five patients presented with learning disabilities and five others exhibited significant behavioural changes. This highlights the importance of gathering such data, although they did not appear as a node in the decision tree.

Among the MSD cases given the wrong predictive diagnosis, we noted several positive symptoms linked to other symptoms. These affected a number of dimensions. Eight cases demonstrated the co-existence of mood-related symptoms [1 to 6] during adolescence. 0 to 3 were more or less negative and inclining spontaneously towards the diagnosis of MSD, albeit with consequently positive symptoms [3 to 8]. Those symptoms are probably more evident and could potentially dominate the clinical picture. A profile therefore emerges combining mood-related symptoms consistent with evolution towards MSD and patent psychotic symptoms presented by less than one-third of the patients in our MSD group.

This atypical profile, at the crossroads of the distinct evolutive trajectories previously described, may be explained by the fact that mania and schizophrenia prodrome characteristics overlapped considerably [21]. Although a clinically high-risk population could be defined, the overlap with the schizophrenia prodrome warrants further investigation and comparison of both illness prodromes. Three subgroups based on pre-morbid adjustment (PMA) were recently identified [30]. PMA measurements include performance during childhood and adolescence in prominent developmental domains such as social and academic functioning [31], using criteria similar to some of the symptoms in our grid. The proportion of SZ and MSD diagnoses, current neurocognition and functioning did not differ between the three identified clusters. These findings are consistent with the common neurodevelopmental hypothesis regarding SZ and MSD, with subgroups exhibiting distinct PMA trajectories that cut across various disorders.

The decision tree could be supplemented and combined with other techniques ranging from a semi-structured interview to genetics [32] via biomarkers [33]. In research, models combining different types of data offer considerable perspectives in personalised medicine [34, 35]. In daily practice, our study could provide targeted diagnostic assistance in documenting medical histories. It could provide clinicians with a global overview of adolescent symptoms combined with the presence of psychosocial factors or a psychiatric family history. This knowledge combined with an awareness of atypical profiles should promote early identification of high-risk SZ or MSD patients, which would facilitate early prevention and effective intervention [11, 36].

### General description and trajectories through childhood and adolescence

Whereas the schizophrenia gender ratio is generally one to one, our SZ population had a 67.5% male component. This can be explained by the time-frame analysed, which did not explore symptoms between the age of 18 and the age of diagnosis. Indeed, the first psychotic episode is usually identified between the age of 15 and 25 in males, and later in females [37]. Moreover, the prodromal phase tends to last 5 years on average [38]. On the contrary, our MSD population mostly comprised female subjects (71.6%) with bipolar disorders affecting men and women to equal extent [39]. Based on a 2021 study involving minors, girls were shown to have better mental health skills than boys of the same age. These skills include emotional regulation, social relationships and empathy, etc. Given their introspection, girls are more capable of asking for and accepting help [40]. We can assume that young girls seek more help during adolescence – hence their over-representation in the study sample.

According to the HAS, the French public health authority, the most interesting age-group for screening is the 15 to 25 year-old age group, which exceeds our timeframe. This information is consistent with the average age of diagnosis in our study: 20.6 (SD=3.9) for the SZ group, 20.9 (SD=4.1) for the MSD group.

The initial psychiatric consultation mostly took place at the end of childhood (at 12.1 years of age on average SD=4.8 for SZ, 11.7 years of age SD=4.5 for MSD), which coincides with the pre-morbid phase. It groups together relatively non-specific symptoms common to the psychotic pre-morbid phase, namely cognitive, anxiety-related symptoms and oppositional behaviours as identified in a meta-analysis [1]. A significant change in behaviour or function was noted in 23.1% of the MSD group at an average age of 14.2 years (SD=2.1). This concept is even more prevalent in the SZ group, affecting 52% of patients with an average age of 14.9 years (SD=1.8). It may correspond to the noisy entry into the prodromal phase alongside puberty.

During the data analysis, a wide range of symptoms appeared in a specific order (Figures 1 and 2). Other studies have also shown that the prodromal phase does not comprise pathognomonic symptoms but a succession of symptoms over time [38, 41]. SZ patients tended to present more difficulties at each developmental stage (neurocognitive deficits, poorer academic and social skills) and ongoing difficulties potentially exacerbated with the emergence of behavioural, negative and positive symptoms. Moreover, bizarre behaviours and the earlier entry point in the decision tree are indicative of positive symptoms (14.7 years old, SD=2.9). Future schizophrenic patients were already described as strange, bizarre children [38, 42]. Patients developing a bipolar disorder were more likely to exhibit anxiety-related symptoms in childhood and mood-related symptoms during adolescence, as shown in the literature [1]. The prevalence of suicidal thoughts is marked in both groups (37.4% at an average age of 15.2 years for SZ and 39% at an average age of 14.8 years for MSD), echoing the continued importance of suicidal risk prevention during the recovery period [43].

Our observations are consistent with a 2017 literature review [16] highlighting the fact that subjects later diagnosed with schizophrenia and, to a lesser extent, those subsequently diagnosed with bipolar disorder, showed signs of pre-morbid abnormalities such as developmental deviations and adjustment problems. Nonetheless, the predictive risk of each isolated developmental marker remains low. We are facing the same issue: the absence of pathognomonic symptoms tending towards schizophrenia or mood spectrum disorder. Thus, some patients experienced their first pre-morbid symptoms during adolescence. During the latter, subjects may display a labile mood because of puberty, but clinicians should pay close attention to these symptoms as they may be indicative of schizophrenia or mood spectrum disorder”.

### Study strengths and limitations

One of our main limitations lies in the retrospective examination of the medical records. Some were based on several consultations with few relevant clinical features and/or a limited window of time, while others were much more evidence-based. This may lead to false negatives in the results presented. Furthermore, it is a well-known fact that decision trees are unstable, i.e. a minor change in the data can lead to a significant change in the structure of the optimal decision tree. In terms of our sample, it is limited by the number of patients diagnosed with schizophrenia or bipolar disorder in the region and the small proportion of subjects with a paediatric psychiatric record. Moreover, we included subjects with F33 and F34 diagnoses who may have another clinical profile. This could explain why there are more incorrect diagnoses in the decision tree for subjects with a mood spectrum disorder (39%). Increasing the size of the cohort and comparing it to a control group could generate greater specificity for the pre-morbid and prodromal phases of each condition. Finally, like all retrospective studies, this particular study is biased. The quality of the data obtained using this method does not equate to that of a prospective study. Finally, only patients with childhood and adolescent psychiatric consultations were enrolled in the study. This study still provides an overview of the distinct trajectories between both populations and the construction of a decision tree. This tool offers several advantages in that it is easy to understand and interpret following a brief explanation. Moreover, it can be combined with other decision techniques.

## Conclusion

The results of this study, which compares the evolution and age of onset of various symptom categories during childhood and adolescence, allowed us to plot separate trajectories for patients developing schizophrenia or mood spectrum disorder. Overall, our results corroborated current literature findings and are consistent with the neurodevelopmental process which exhibits pre-morbid and prodromal phases. Patients with schizophrenia during adulthood tended to present more difficulties at each developmental stage such as neurocognitive deficits and poorer academic and social skills. These difficulties persist and, in some instances, are exacerbated with the emergence of negative and positive behavioural symptoms. Patients with mood spectrum disorder during adulthood are more likely to have exhibited anxiety-related symptoms in childhood and mood-related symptoms during adolescence. The frequency of suicidal thoughts was substantial in both groups, which emphasises the importance of suicidal risk prevention during the recovery period [43]. Nevertheless, the symptoms were found to be non-pathognomonic during the clinical course of both disorders. We succeeded in extracting a decision tree based on variables associated with one diagnosis. It offered good predictability in our population: 77.6% of patients received a well-indexed diagnosis. We noticed an atypical profile in future bipolar patients as some exhibited numerous positive symptoms combined with more conventional mood-related symptoms. The combination of all these data could be instrumental in the early identification of high-risk patients, thereby facilitating early prevention and intervention [25]. Indeed, certain intervention strategies may be proposed in the early stages of disorder progression. For example, in the case of schizophrenia, subjects at high risk of clinical psychosis [44] can benefit from programmes based on remediation, namely “the Recognition and Prevention (RAP) programme” [45]. Other early interventions are available based on cognitive-behavioural therapy such as cognitive insight improvement, stress management and family focus, etc. and for the treatment of comorbidities (anxiety and depression), as recommended by the European Psychiatry Association [46–48]. These treatment options are increasingly available in routine clinical practice through specific centres [10, 11].

Fewer studies are available for subjects with mood spectrum disorders. However, it is still crucial to make the correct diagnosis in the early stages and offer specific care in the form of psychological education, stress management and addiction prevention, as recommended for a number of serious mental illnesses [49].

Moreover, transdiagnostic studies tend to emerge in the scientific literature, sometimes challenging conventional Kraepelinian dichotomy [50], sometimes assuming a continuum between bipolar disorders and schizophrenia. A number of domains are affected, such as genetics and neurotransmitters [32, 51, 52], neuro-imagery [53, 54] with a retinal focus [55, 56], immunology and microbiome [57], cognitive evaluation and pre-morbid adjustment [30, 58]. Some authors advocate a less categorial, more dimensional study of psychiatric illness, proposing models such as the RDoC (Research Domain Criteria) [59] or, more specifically, ESSENCE (Early Symptomatic Syndromes Eliciting Neurodevelopmental Clinical Examinations), which suggest the co-existence of disorders and pooling of symptoms across disorders as the rule rather than the exception [60]. The global expectation is that the identification of neurophysiology-based syndromes will eventually lead to improved outcomes.

## Data Availability

Material (data grid used for the data collection) and data are available by contacting the correspondence author.

## Abbreviations

MSD: Mood Spectrum Disorder
SZ: Schizophrenia
